# Monitoring tumor response to the vascular disrupting agent CKD-516 in a rabbit VX2 intramuscular tumor model using PET/MRI: Simultaneous evaluation of vascular and metabolic parameters

**DOI:** 10.1371/journal.pone.0192706

**Published:** 2018-02-13

**Authors:** Su Yeon Ahn, Jin Mo Goo, Kyung Hee Lee, Seunggyun Ha, Jin Chul Paeng

**Affiliations:** 1 Department of Radiology, Seoul National University College of Medicine, and Institute of Radiation Medicine, Seoul National University Medical Research Center, Seoul, Korea; 2 Cancer Research Institute, Seoul National University, Seoul, Korea; 3 Department of Radiology, Seoul National University Bundang Hospital, Seongnam-si, Gyeonggi-do, Korea; 4 Department of Nuclear Medicine, Seoul National University College of Medicine, Seoul, Korea; Northwestern University Feinberg School of Medicine, UNITED STATES

## Abstract

**Objectives:**

To determine whether the CKD-516 produces a significant change in vascular and metabolic parameters in PET/MRI

**Materials and methods:**

With institutional Animal Care and Use Committee approval, 18 VX2 carcinoma tumors implanted in bilateral back muscles of 9 rabbits were evaluated. Serial PET/MRI were performed before, 4 hours after and 1-week after vascular disrupting agent, CKD-516 at a dose of 0.7 mg/kg (treated group, n = 10) or saline (control group, n = 8) administration. PET/MRI-derived parameters and their interval changes were compared between the treated and control group by using the linear mixed model. Each parameter within each group was also compared by using the linear mixed model.

**Results:**

Changes of the volume transfer coefficient (K^trans^) and the initial area under the gadolinium concentration-time curve until 60 seconds (iAUC) in the treated group were significantly larger compared with those in the control group at 4-hour follow-up (mean, -39.91% vs. -6.04%, P = 0.018; and -49.71% vs. +6.23%, P = 0.013). Change of metabolic tumor volume (MTV) in the treated group was significantly smaller compared with that in the control group at 1-week follow-up (mean, +118.34% vs. +208.87%, P = 0.044). Serial measurements in the treated group revealed that K^trans^ and iAUC decreased at 4-hour follow-up (P < 0.001) and partially recovered at 1-week follow-up (P = 0.001 and 0.024, respectively). MTV increased at a 4-hour follow-up (P = 0.038) and further increased at a 1-week follow-up (P < 0.001), while total lesion glycolysis (TLG) did not show a significant difference between the time points. SUVmax and SUVmean did not show significant interval changes between time points (P > 0.05).

**Conclusions:**

PET/MRI is able to monitor the changes of vascular and metabolic parameters at different time points simultaneously, and confirmed that vascular changes precede the metabolic changes by VDA, CKD-516.

## Introduction

Supply of oxygen and nutrients via the surrounding vasculature is essential for tumor growth. Therefore, tumor vasculature has been a major target for cancer treatment. Apart from antiangiogenic drugs which compromise the formation of new blood vessels, vascular disrupting agents (VDAs) target the established tumor vasculature and cause a shutdown of blood flow, leading to subsequent tumor ischemia and necrosis [1]. Because of its cytostatic nature, conventional assessment of tumor response based on reduction in tumor size, using Response Evaluation Criteria in Solid Tumors [2] may not be adequate or prompt since there is a significant latency in determining the clinical effectiveness [3]. Physiologic or metabolic responses occur soon after the start of anticancer therapy, although clinical responses are slow. Therefore, functional molecular imaging techniques that depict physiologic and cellular processes within tumors such as vascularity or metabolism have been emphasized [4]. The depiction of these post-therapeutic events earlier than clinical endpoint is helpful for choosing the right treatment strategy, preventing unnecessarily long treatment courses with their inherent adverse events, as well as deciding whether to go or not in the development of anticancer pharmaceuticals [5, 6]. Several imaging techniques have been investigated to assess angiogenic vasculature and monitor the efficacy of vascular targeting agents noninvasively [3]. The majority of them were studied with dynamic contrast-enhanced magnetic resonance imaging (DCE-MRI) parameters for assessing the therapeutic effect of vascular targeting agents [3, 6–10]. Furthermore, only a few previous studies were performed with [^18^F]-fluorodeoxyglucose (FDG) positron emission tomography (PET) for assessing metabolic change after the therapeutic effect of anti-vascular drugs [11–13]. However, to our knowledge, until now, no studies dealing with post-treatment changes after VDA have been performed yet with the novel imaging modality PET/MRI, which can obtain multiparametric information simultaneously.

Vascular disrupting agent, CKD-516 used in this experiment is a tubulin polymerization inhibitor that has a dual action mechanism of (a) rapid disruption of pre-existing tumor vasculature, resulting in hypoxia and necrosis, and (b) arresting the cell cycle, resulting in apoptosis [14].

Therefore, the aim of this study is to determine whether the CKD-516 produces a significant change in vascular and metabolic parameters in PET/MRI.

## Materials and methods

### Ethics statement

All experiments were approved by the Institutional Animal Care and Use Committee in Seoul National University Hospital (SNUH-IACUC) (Permit Number: 15-0241-S1A0(1)) and animals were maintained in the facility accredited AAALAC International (#001169) in accordance with Guide for the Care and Use of Laboratory Animals 8th edition, National Research Council (2010). The rabbits were monitored every 1–2 days. If one of the following conditions occurred during the experiment, the animals were humanely killed considered: weight loss (>20%) or if the animal became cachectic, had difficulty eating, drinking or moving around freely. All efforts were made to minimize suffering of animals during tumor implantation and image acquisition.

### Animal model

Sixteen male New Zealand White rabbits (Koatech Co. Ltd., Pyongtaek, Korea) weighing 3.0–3.5 kg were used. The rationale for choosing rabbit VX2 model is as follows; rabbit VX2 tumor model is well established for studies of tumor and histopathological features are similar to humans [15], thus the results of animal studies can be extrapolated to humans; many previous studies have used the rabbit VX2 model for functional imaging [9, 16]; the VX2 carcinoma strain had been maintained by continuous transplantation into the hind limbs of carrier rabbits at the preclinical animal experimental center of our institute, making it easily accessible in this study. To help the rabbits adapt to their environment, they were housed in individual cages with freely available food and water for 1–2 weeks in accordance with our humane animal care protocol. For each rabbit, anesthesia was induced with intravenous ketamine hydrochloride (50 mg per kg of body weight; Ketamine, Yuhan, Korea) and 2% xylazine (0.1 mL/kg; Rompun, Bayer, Germany).

After anesthesia, bilateral back parallel to the spine was shaved and sterilized. Thereafter, 0.2 ml of VX2 tumor suspension was slowly implanted symmetrically in the bilateral paravertebral muscles at the level of the kidney each using 18-gauge Chiba needle under the guidance of ultrasonography (Accuvix XG, Samsung Medison, Seoul, Korea). Tumors were incubated for 10–12 days after the tumor implantation prior to baseline imaging. Among the 16 rabbits, the total number of tumors was 32. The longest diameter of the tumors on baseline T2-weighted axial MR images was 16.2 ± 3.5 mm (range, 9.3–22.2 mm).

### Experimental protocol

Sixteen rabbits were randomly allocated to receive injections of CKD-516 (Chong Kun Dang Pharmaceutical, Seoul, Korea) at a dose of 0.7 mg/kg (n = 11) or normal saline (n = 5) (treated group and control group, respectively). We set the dose of CKD-516 according to the safe dose in human (9 mg/m^2^) and converted it to the equivalent dose in rabbits (0.75mg/kg) [9]. One day after the baseline imaging, CKD-516 diluted in 3 mL of normal saline was administered slowly at a rate of 1 mL/min via the auricular vein to the treated group and the same dose of normal saline to the control group. Follow-up imaging was performed 4 hours and 1-week after the administration. After the last PET/MR imaging session, all rabbits were sacrificed by intravenous injection of 5 ml potassium chloride while under deep anesthesia.

### PET/MRI image acquisition

All acquisitions were performed by using an integrated PET/MRI scanner (Biograph mMR, Siemens Healthcare, Erlangen, Germany). All animals were fasted for at least 6 hours prior to the PET/MRI examination. Animals had an intravenous access established in the auricular vein at the day of PET/MRI acquisition. FDG (37 MBq) was intravenously injected and 1-bed PET image was obtained for 10 min, approximately 60 min after injection. MR acquisition was initiated as soon as the animals were placed in a supine position in the scanner. Using three-plane, true-fast imaging with steady-state precession (true-FISP) localizers, an axial MRI slab was placed covering the whole tumor implant in paravertebral muscles. T2-weighted images (T2WI) were obtained with the following parameters: repetition time/echo time (TR/TE), 4230/84 ms; matrix size, 128×128; slice thickness, 3 mm; and field of view (FOV), 160×160 mm. Unenhanced T1-weighted volumetric interpolated breath-hold examination (VIBE) images were acquired at each flip angle for T1 mapping using the following parameters: TR/TE 3.2/1.1 ms; flip angles (α = 2°, 5°, 10° and 15°); matrix size 128×128; slice thickness 3 mm; number of slices 20; and FOV 160 mm. Thereafter, dynamic contrast-enhanced MR imaging using the T1-weighted radial gradient echo sequence was then performed after an intravenous bolus injection via the auricular vein of 0.1 mmol/kg of gadoteric acid (Dotarem; Guerbet, Paris, France). DCE-MRI using the VIBE sequence was obtained at 5 seconds of temporal resolution and the parameters were TR/TE 3.2/1.1 ms, flip angles (α = 15°), matrix size 128×128, slice thickness 3 mm, number of slices 20, and FOV 160 mm. The total acquisition time of the dynamic scan was 10 minutes, including the first 4 phases of pre-contrast images.

During MRI acquisition, emission data were collected from a single bed position of tumor implant level for 20 minutes and list-mode dataset was acquired for all animals. Reconstruction of PET images was performed on the mMR console using 3D-ordered subset expectation maximization (3D-OSEM) with point spread function modeling with 3 iterations, 21 subsets, image matrix 256, a zoom factor of 2.

### Image analysis

The most commonly used two DCE-MRI parameters, the volume transfer coefficient (K^trans^) and the initial area under the gadolinium concentration-time curve until 60 seconds (iAUC) were measured, according to the consensus opinion on DCE-MRI in evaluating vascular targeting agents in previous studies [1, 7, 17]. Using the DCE-MRI images, parametric maps of K^trans^ and iAUC were generated with a post-processing software program (Tissue4D; Siemens Medical Solutions) based on Tofts model [18, 19]. We selected a representative section with the longest diameter of tumor and drew a region of interest manually by outlining the entire tumor boundary.

The maximum and average standardized uptake value (SUVmax and SUVmean) were measured to determine FDG avidity of the tumors using commercial software (Syngo.via, Siemens Healthcare). A sphere-shaped volume of interest (VOI) that included the entire lesion was drawn to determine FDG avidity. Metabolic tumor volume (MTV) was defined as total tumor volume with a margin threshold of 40% SUVmax. Total lesion glycolysis (TLG) was calculated as (SUVmean) × (MTV) in the same isocontour VOI.

Tumor size was defined as the longest diameter measured on axial T2-weighted images. In addition, tumor volume was measured using a semi-automatic segmentation tool. Percentage changes in PET/MRI-derived parameters relative to the baseline were calculated as follows: Value Change = (Value_given time_-Value_baseline_)/Value_baseline_ × 100%.

### Statistical analysis

To determine whether there were differences in tumor size, volume, PET/MRI imaging values and their interval changes between the treated and control groups, the linear mixed model was used which made it possible to analyze clustered data because each rabbit has two tumors. Two fixed effects were included: one between-subjects group (control vs. treated) effect and one within-subject time effect (time: baseline, 4 hours, and 1 week). Spearman rank correlation test was performed to evaluate the correlation between the changes in PET/MR imaging values at each time point compared with the baseline. A P value of less than 0.05 was considered to indicate a significant difference. All statistical analyses were performed with SPSS version 21 (SPSS, Chicago, IL, USA).

## Results

Among 16 rabbits, data from 3 rabbits (2 in the treated group and 1 in the control group) were incomplete because 1-week follow-up imaging was not available due to technical problems. In addition, 4 rabbits in the treated group died unexpectedly before humane endpoints, mostly one or two days before 1-week follow-up. Although the dose of CKD-516 was within safe limits and we did not investigate the toxicity in our study, the toxicity may be related to animal’s death because all dead rabbits were in the treated group. However, stressful situation such as repeated handling, transportation and anesthesia may also be related. Therefore, 18 tumors in 9 rabbits (10 tumors in 5 rabbits in the treated group and 8 tumors in 4 rabbits in the control group) were available for all baselines, 4 hours and 1-week follow-up.

### Sequential change in size and volume of tumor

The size of tumors at baseline in the control and treated groups was 18 ± 4 mm and 16 ± 4 mm, respectively, and it showed no significant differences (*P >* 0.05). At 1-week follow-up, the tumors grew to 22 ± 3 mm and 20 ± 5 mm, respectively. Percentage change of size of tumors at 1-week follow-up did not show a significant difference between the two groups (30.9% vs. 21.9%, *P* = 0.198).

The volume of tumors at baseline in the control and treated group was 6.0 ± 2.9 cm^3^ and 4.6 ± 2.3 cm^3^, respectively, and it showed no significant differences (*P* = 0.438). At 1-week follow-up, the tumors grew to 13.5 ± 5.3 cm^3^ and 8.1 ± 3.1 cm^3^, respectively. Percentage change of volume of tumors at 1-week follow-up showed no significant differences between the treated and control groups (139.3% vs. 89.6%, *P* = 0.115).

### Comparison of PET/MRI parameters at each time points and their changes between the treated and control groups

The time × group interaction effect was found in K^trans^, iAUC, MTV and TLG (*P* = 0.023, <0.001, 0.001 and <0.001, respectively) on linear mixed model analysis. Mean values of vascular and metabolic parameters and comparison of each parameter between treated and control group at each time points and comparison of each parameter between time points in each group are shown in Table 1. Percentage changes of those relative to baseline for each group at each time point are summarized in Table 2.

**Table 1:** Comparison of vascular and metabolic parameters between the control and treated groups at each time point and comparison of each parameter between time points in each group.

	Control (n = 8)	P value		Treated (n = 10)	P value		P value
		4 Hours	1 Week		4 Hours	1 Week	
**K**^***trans***^ **(min**^**-1**^**)**							time × group: 0.023
Baseline	0.29 ± 0.13	0.137	0.049	0.28 ± 0.69	<0.001	0.001	0.816
4 Hours	0.26 ± 0.08		0.062	0.16 ± 0.05		0.081	0.055
1 Week	0.19 ± 0.06			0.20 ± 0.07			0.903
**iAUC (mmol/sec)**							time × group: <0.001
Baseline	27.85 ± 10.39	0.853	0.037	26.49 ± 5.82	<0.001	0.024	0.819
4 Hours	28.31 ± 8.30		0.014	12.95 ± 5.44		<0.001	0.015
1 Week	18.58 ± 5.22			21.23 ± 7.76			0.555
**SUVmax**							time × group: 0.143
Baseline	8.72 ± 2.91	NA	NA	6.44 ± 2.00	NA	NA	NA
4 Hours	6.91 ± 3.01		NA	5.77 ± 1.80		NA	NA
1 Week	8.79 ± 2.70			5.52 ± 2.59			NA
**SUVmean**							time × group: 0.237
Baseline	5.50 ± 2.02	NA	NA	4.00± 1.22	NA	NA	NA
4 Hours	4.37 ± 1.91		NA	3.50 ± 1.14		NA	NA
1 Week	5.21 ± 1.59			3.19 ± 1.56			NA
**MTV (cm**^**3**^**)**							time × group: 0.001
Baseline	3.78 ± 2.01	0.018	<0.001	2.81 ± 1.09	0.038	<0.001	0.374
4 Hours	4.65 ± 2.00		<0.001	3.66 ± 0.91		0.005	0.345
1 Week	10.69± 4.02			6.09 ± 2.17			0.035
**TLG**							time × group: <0.001
Baseline	23.96 ± 21.28	0.627	0.001	10.66 ±3.64	0.341	0.066	0.225
4 Hours	21.67 ± 13.47		0.001	12.41 ± 4.33		0.126	0.193
1 Week	60.23 ± 35.20			18.93 ± 12.85			0.035

Data are presented as mean ± standard deviation

K^trans^: volume transfer coefficient

iAUC: initial area under the gadolinium concentration-time curve until 60 seconds

SUV: standardized uptake value

MTV: metabolic tumor volume

TLG: total lesion glycolysis

**Table 2:** Percentage changes of vascular and metabolic parameters compared with the baseline at each time point and P values of comparisons between the treated and control groups.

	Control (n = 8)	Treated (n = 10)	*P* Value
**K**^**trans**^ **(min**^**-1**^**)**			
4 Hours	-6.04(-21.04, 8.97)	-39.91(-52.64, -27.18)	0.018
1 Week	-29.90(-66.07, 6.27)	-27.35(-45.62, -9.08)	0.822
**iAUC (mmol/sec)**			
4 Hours	6.23(-17.75,30.21)	-49.71(-65.47, -33.95)	0.013
1 Week	-24.15(-53.84,5.55)	-18.87(-38.17, 0.43)	0.797
**SUVmax**			
4 Hours	-15.57(-52.41, 21.27)	-7.97(-25.83, 9.89)	0.757
1 Week	4.89(-23.17, 32.95)	-16.42(-35.03, 2.18)	0.299
**SUVmean**			
4 Hours	-14.82(-51.87, 22.23)	-10.05(-30.41, 10.30)	0.852
1 Week	-0.76 (-27.03, 25.51)	-21.92(-42.63, -1.21)	0.303
**MTV (cm**^**3**^**)**			
4 Hours	30.74(14.05, 47.72)	37.82(16.61, 59.03)	0.660
1 Week	208.87(145.75, 272.00)	118.34(76.59, 160.10)	0.044
**TLG**			
4 Hours	195.96(129.96, 261.97)	42.23(2.68, 81.77)	0.623
1 Week	209.64(108.00, 311.28)	79.43(3.74, 155.14)	0.101

Data are relative percentage changes determined by comparing the value at baseline with that at follow-up. Data in parentheses are 95% confidence intervals.

K^trans^: volume transfer coefficient

iAUC: initial area under the gadolinium concentration-time curve until 60 seconds

SUV: standardized uptake value

MTV: metabolic tumor volume

TLG: total lesion glycolysis

There were no significant differences in all of PET/MRI parameters at baseline imaging between the two groups. At 4-hour follow-up, K^trans^ in the treated group were lower than that in the control group, although the difference showed no statistical significance (*P* = 0.055). iAUC in the treated group were significantly lower than that in the control group (*P* = 0.015) at 4-hour follow-up. At 1-week follow-up, MTV and TLG were significantly lower in the treated group than those in the control group (*P* = 0.035).

Percentage changes of PET/MRI parameters were also evaluated by using the linear mixed model. Changes of K^trans^ and iAUC in the treated group were significantly larger compared with those in the control group at 4-hour follow-up (mean, -39.91% vs. -6.04%, *P* = 0.018; and -49.71% vs. +6.23%, *P* = 0.013). None of the relative changes in PET-derived parameters showed any statistically significant differences between the treated and control groups at 4-hour follow-up.

MTV in the treated group is significantly less increased compared with those in the control group at 1-week follow-up (mean, +118.34% vs. +208.87%, *P* = 0.003). No statistical differences were observed in percentage changes in SUVmax, SUVmean, TLG, K^trans^ and iAUC between the two groups at 1-week follow-up.

### Sequential change in PET/MRI parameters of tumor within each group

**Fig 1:**
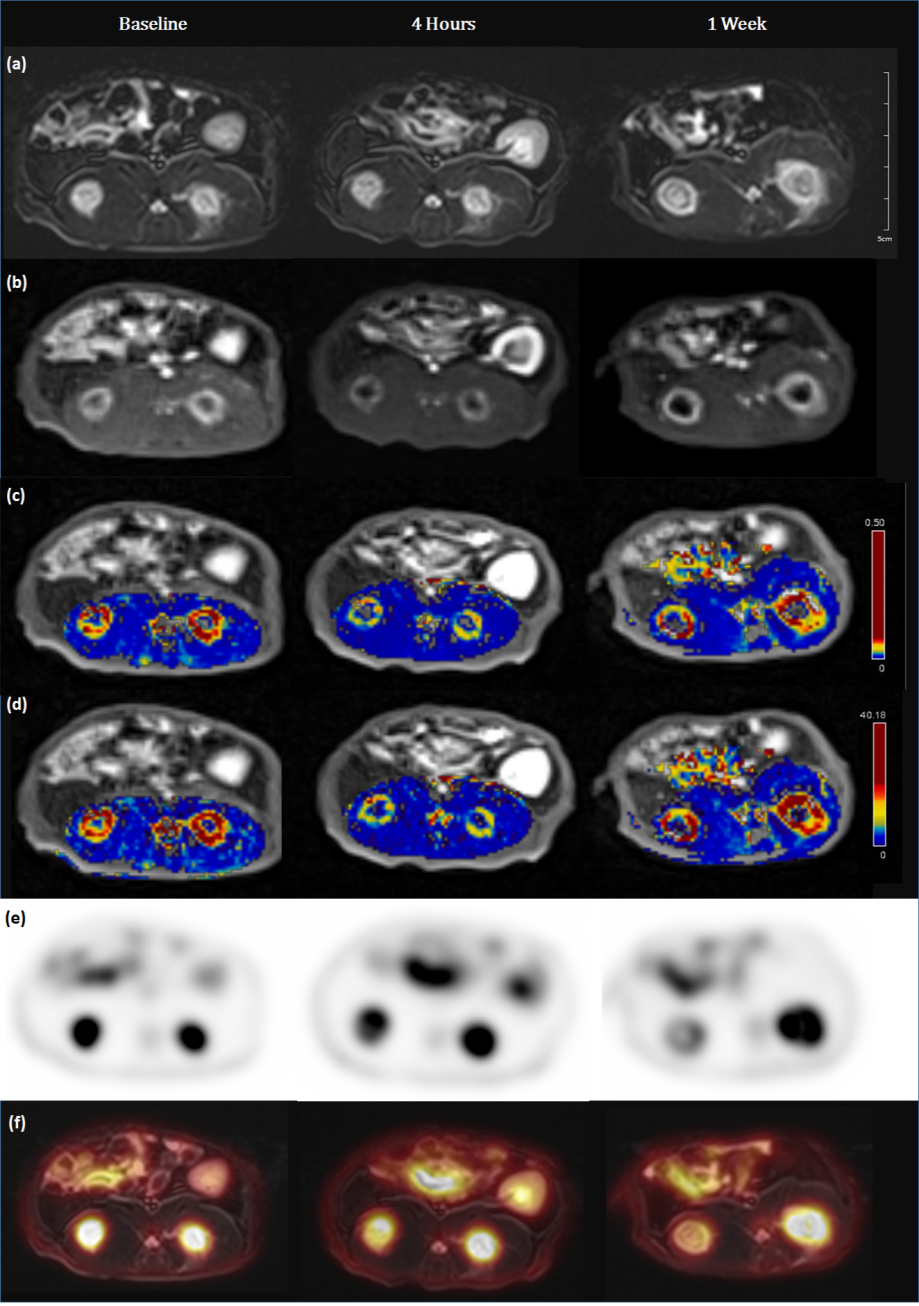
Serial changes of PET/MRI parameters before and after the treatment with CKD-516. (a) Axial T2-weighted images demonstrated target tumors with high signal intensity in bilateral paravertebral muscles. (b) DCE-MRI revealed peripheral enhancement of tumors suggesting central necrosis. (c) K^trans^ map and (d) iAUC map demonstrated a reduction of values at 4 hours and partial recovery at 1 week follow-up. (e) PET images and (f) fusion images showed no significant differences of SUVmax or SUVmean.

**Fig 2:**
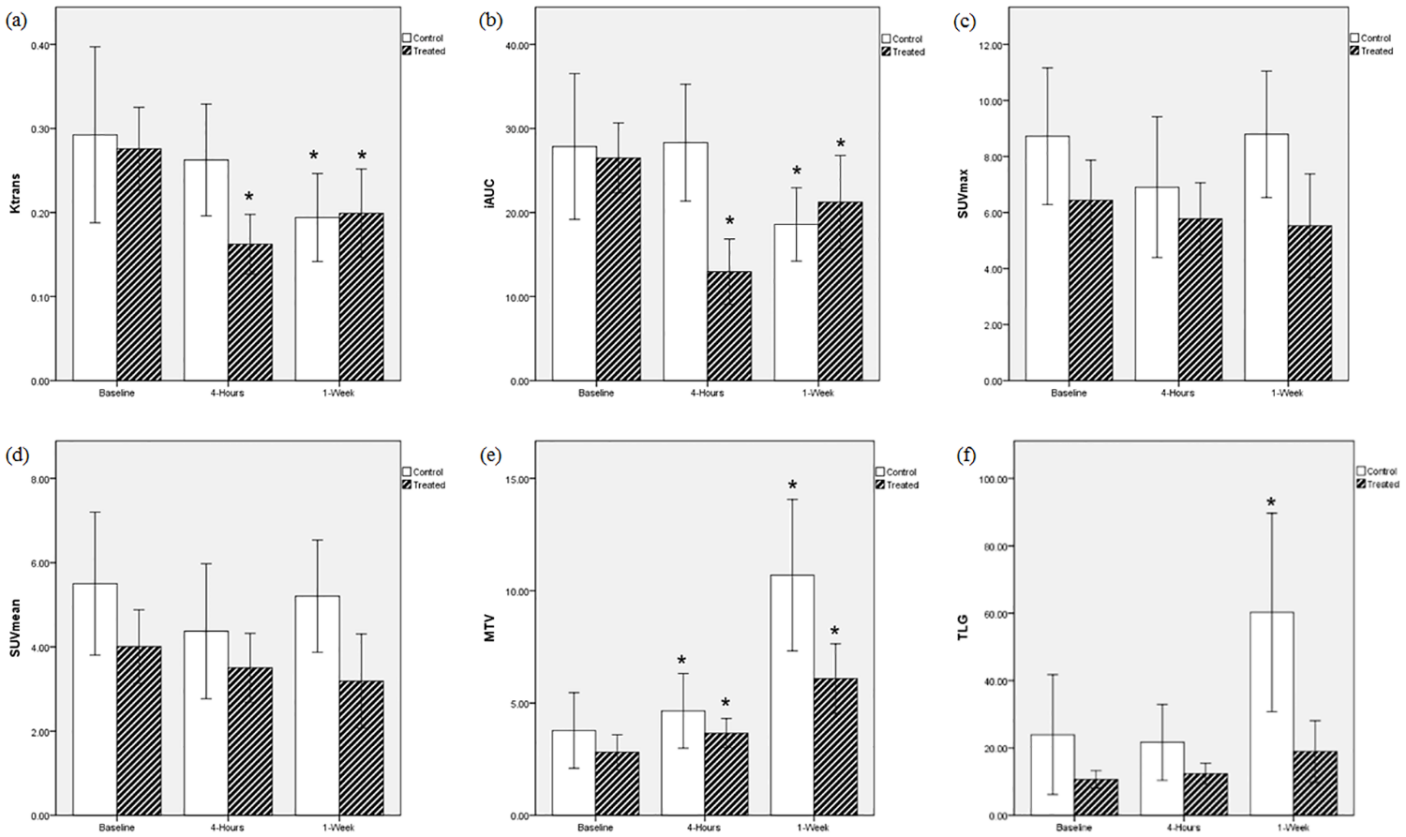
Serial measurement of (a) K^trans^, (b) iAUC, (c) SUVmax, (d) SUVmean, (e) MTV and (f) TLG at different time points. * is a significant change compared to the baseline. K^trans^: volume transfer coefficient iAUC: initial area under the gadolinium concentration-time curve until 60 seconds SUV: standardized uptake value MTV: metabolic tumor volume TLG: total lesion glycolysis.

Comparisons between different time points in each parameter are shown in Figs 1 and 2. Serial measurements in the treated group revealed that K^trans^ and iAUC decreased at 4-hour follow-up (*P <* 0.001) and partially recovered at 1-week follow-up (*P* = 0.001 and 0.024, respectively). MTV increased at 4-hour follow-up (*P* = 0.038) and further increased at 1-week follow-up (*P <* 0.001), while TLG increased at 1-week follow-up without a significant difference (*P* > 0.05).

In the control group, K^trans^ and iAUC showed no significant differences at 4-hours follow-up (*P* > 0.05) and decreased at 1-week follow-up (*P* = 0.049 and 0.037, respectively). MTV increased at 4-hour follow-up (*P* = 0.018) and further increased at 1-week follow-up (*P <* 0.001), while TLG showed no significant difference at 4-hour follow-up and increased at 1-week follow-up (*P* = 0.001) in the control group.

### Correlation analysis with tumor response

To determine whether there is correlation between the percentage changes in parameters at 4-hour follow-up and change of tumor size or volume at 1-week follow-up for early prediction of tumor response, correlation analysis was performed in the treated group. Any change of parameters at 4-hour follow-up was not correlated with the change of size or volume at 1-week follow-up. (*P>* 0.05). In addition, we have checked the correlations between vascular and metabolic parameters (K^trans^ or iAUC and 4 metabolic parameters, respectively) and we’ve found that there were no correlation between these parameters at any time (p>0.05).

## Discussion

Our study demonstrated that PET/MRI can monitor serial vascular and metabolic changes of intramuscular VX2 tumor model after the administration of VDA, CKD-516. Vascular and metabolic parameters changed at different times and we confirmed that vascular changes precede the metabolic changes by VDA, CKD-516. Significant differences in vascular parameters including K^trans^ and iAUC occurred after 4 hours, whereas significant differences in metabolic parameters of MTV and TLG were noted at 1-week after VDA treatment between the control and treated groups.

**Fig 3:**
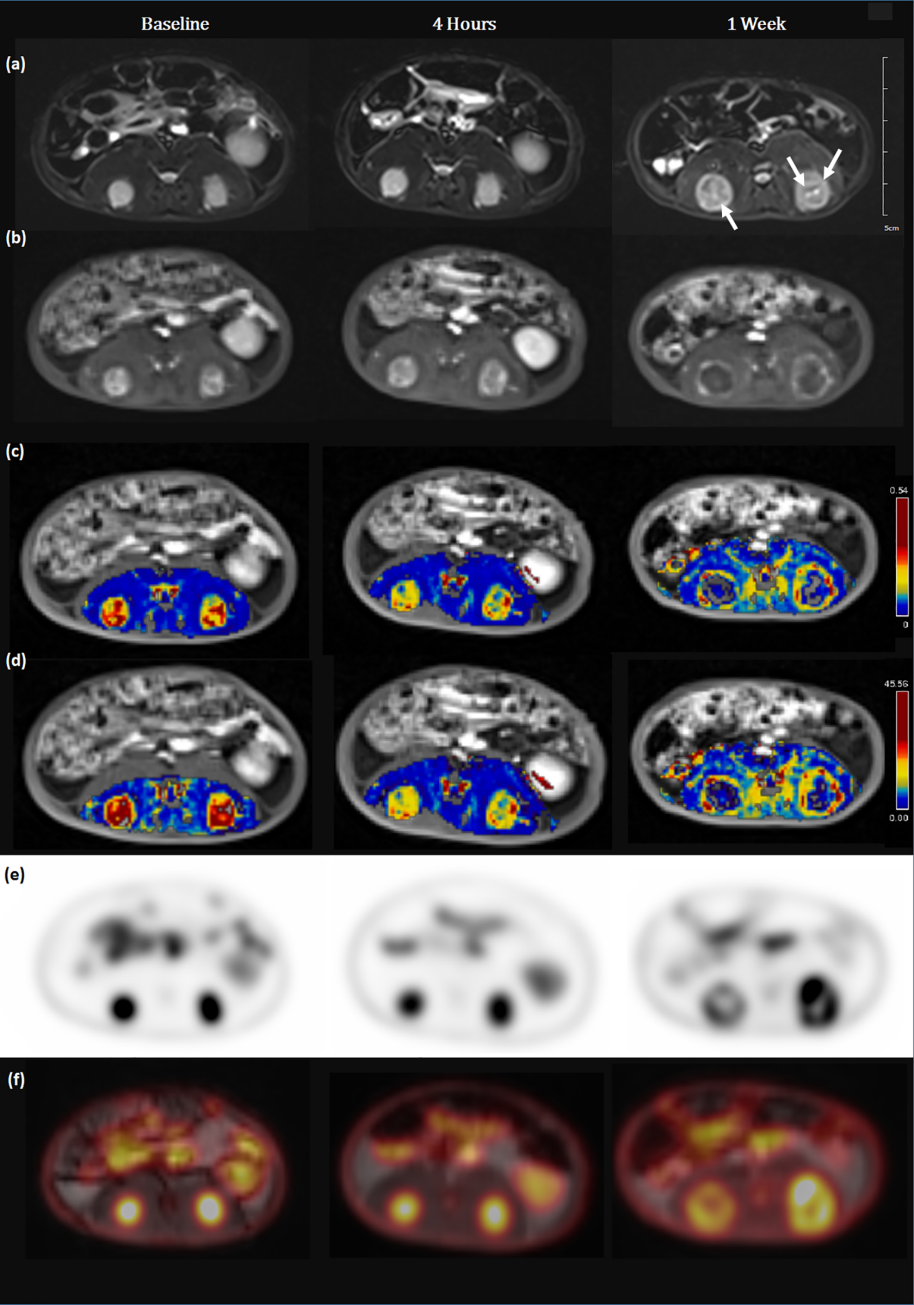
Serial changes of PET/MRI parameters in control group. (a) Axial T2-weighted images demonstrated target tumors with high signal intensity in bilateral paravertebral muscles. At 1-week follow-up, there were areas of high signal intensity within tumors on T2 weighted images, suggestive of necrosis (arrows). (b) DCE-MRI, (c) K^trans^ map, (d) iAUC, (e) PET images and (f) fusion images.

Our results of vascular parameters are similar to previous studies that have demonstrated that K^trans^ and iAUC values changed within a few hours after the treatment with VDA [7–9, 20]. Both parameters reflect blood flow and permeability [3]. Tumor blood vessels are immature and highly permeable without supporting connective tissues, and VDA perturbs these preexisting vessels, leading to collapse of tumor vasculature and subsequent necrosis [21]. Thus, a decrease of K^trans^ and iAUC reflects a decrease in blood flow and permeability in a tumor. Interestingly, serial measurement of Ktrans and iAUC in control group also showed significant decrease at 1 week follow-up in our study. One of our hypothesis is that it is due to the nature of VX2 tumors. VX2 tumor grows quickly in the first three week and showed necrosis and cysts at advanced stages [22]. In our study, at 1-week follow-up, control group as well as treated group also showed areas of high signal intensity within tumors on T2 weighted images, suggestive of necrosis (Fig 3). According to Moon et al. [23]’s study, K^trans^ value of complete necrotic area is lower than that of partial necrotic area, and K^trans^ value of partial necrotic area is lower than that of viable tumor area. It suggests that necrosis can affect the value of K^trans^. Therefore, we assume that necrotic portion of tumors in control group at 1 week follow-up could cause the decrease of Ktrans. However, K^trans^ and iAUC of baseline and 1 week follow-up did not show significant difference between the treated and control group. Significant differences of values and its changes at 4 hours are important issue in discriminating physiological response to therapy.

Although histologic examination is not available in this study, there were several studies which investigated the correlation of histologic parameters such as necrosis and microvessel density with changes in DCE-MRI parameters. Necrosis can affect the value of K^trans^ [23], which would explain our results of changes of K^trans^. For the microvessel density, there is a controversy whether it could be a validation biomarker for effect of VDA. Because many counted microvessels collapsed 4 hours after CKD-516, microvessel density could not represent the functional vascular profile [9].

Despite the rapid shutdown of tumor vasculature, we could not identify the correlation between changes of vascular parameters and tumor response. The potential mechanism is tumor resistance to VDA. In hypoxic condition induced by VDA, tumors upregulate the expression of hypoxia-inducible factor-1 alpha, which involves in angiogenesis, glycolysis, and microenvironment acidification of tumors [24]. Although VDA induces tumor necrosis, viable tumors at the peripheral rim adapt to hypoxia by providing nutrients through newly formed vessels and promote growth and proliferation. El-Emir et al. [25] demonstrated the effect of combretastatin A-4 phosphate, one of VDA, in colorectal xenograft model and revealed that hypoxia reaches its maximum 1 hour and is relieved and returned to normal after 24 hours. Increase of K^trans^ reflects tumor resistance to VDA, recovering tumor perfusion and relapse of tumors [24, 26]. Thus, rapid decrease of perfusion parameters might not be directly associated with size reduction, as shown in our results.

In addition to vascular parameters, we also demonstrated simultaneous changes of metabolic parameters after the administration of CKD-516. Among PET-derived parameters, MTV and TLG showed significant differences at 1-week follow-up between the two groups. Furthermore, an increase in MTV at 1-week follow-up in the treated group was smaller than in the control group. MTV and TLG are potential biomarkers for predicting response to treatment as well as for predicting prognosis in various solid tumors, including head and neck cancer, lung cancer, esophageal cancer, cervical cancer, epithelial ovarian cancer, etc [27]. However, we could not identify prognostic or predictive factors for tumor response in this experiment. None of the changes of parameters including MTV and TLG at 4 hours follow-up was correlated with changes in tumor size at 1-week follow-up. This may be because the follow-up period was too short to cause a change of size.

To our knowledge, this is the first study that depicts multiparametric monitoring of tumor response after the administration of VDA, using the novel imaging technique, PET/MRI. Multimodality imaging is a rapidly growing field in clinical practice, but most are performed on separate machines, which requires time-consuming processing and manipulating a vast amount of imaging. Also, interpreting images side-by-side results in diagnostic inaccuracy [28]. Recently, the potential of simultaneous acquisition of in vivo functional information with PET/MRI has been investigated [16]. We believe that simultaneity is one of the advantages of PET/MR imaging in comparison with separate PET and MRI. Simultaneous acquisition provides better alignment quality and is especially beneficial when temporally varying multi-functional information from PET and MRI are acquired [29]. Although, there is no comparable data acquiring vascular and metabolic parameters separately and simultaneously, we believe that simultaneous PET/MR acquisition is suitable for monitoring multi parametric changes after VDA treatment because they changes at different time points as shown in our study.

Our study has several limitations. First, we conducted baseline imaging 1 day before the administration of CKD-516 considering the elimination of previously injected FDG. Because the VX2 tumor is highly aggressive and grows rapidly [30], the microenvironment of the tumor would change within a day. Thus, changes of parameters at 4 hours after the treatment may include not only the effect of drugs but also an intrinsic change of tumor characteristics. Second, DCE-MRI parameters are measured with one axial cross-sectional image, which might be less representative of the entire tumor. Third, it is not validated whether early changes in vascular and metabolic parameters in PET/MRI after the treatment with VDA are relevant to clinical outcome such as overall survival, so further studies must be performed. Forth, the histopathologic examination or quantification is not available to support the changes of vascular and metabolic of parameters. Although several studies dealing with vascular parameters have provided the histologic correlations, further studies on correlation between the histologic examination and PET parameters are warranted.

In conclusion, PET/MRI is able to monitor the change of vascular and metabolic parameters at different time points simultaneously and confirmed that vascular changes precede the metabolic changes by VDA, CKD-516.

## Supporting information

S1 File Dataset for sequential changes in PET/MRI parameters of all rabbits. (XLSX)

S2 File Animal Research: Reporting In Vivo Experiments (ARRIVE) guidelines checklist. (DOCX)

## Abbreviations

DCE-MRI: dynamic contrast-enhanced magnetic resonance imaging
FDG: fluorodeoxyglucose
iAUC: the initial area under the gadolinium concentration-time curve until 60 seconds
K^trans^: the volume transfer coefficient
MTV: metabolic tumor volume
PET: positron emission tomography
SUVmax: the maximum standardized uptake value
SUVmean: the average standardized uptake value
TLG: total lesion glycolysis
VDA: vascular disrupting agent
